# Yet another job for the bacterial ribosome

**DOI:** 10.15698/mic2019.11.698

**Published:** 2019-10-17

**Authors:** Andrea Origi, Ana Natriashivili, Lara Knüpffer, Clara Fehrenbach, Kärt Denks, Rosella Asti, Hans-Georg Koch

**Affiliations:** 1 Institute of Biochemistry and Molecular Biology, ZBMZ, Faculty of Medicine, Albert-Ludwigs University Freiburg, 79104 Freiburg, Germany.; 2 Faculty of Biology, Albert-Ludwigs University Freiburg, 79104 Freiburg, Germany.

**Keywords:** SecA, signal recognition particle, SecYEG, protein targeting, uL23, ribosome

## Abstract

The ribosome is a sophisticated cellular machine, composed of RNA and protein, which translates the mRNA-encoded genetic information into protein and thus acts at the center of gene expression. Still, the ribosome not only decodes the genetic information, it also coordinates many ribosome-associated processes like protein folding and targeting. The ribosomal protein uL23 is crucial for this coordination and is located at the ribosomal tunnel exit where it serves as binding platform for targeting factors, chaperones and modifying enzymes. This includes the signal recognition particle (SRP), which facilitates co-translational protein targeting in pro- and eukaryotes, the chaperone Trigger Factor and methionine aminopeptidase, which removes the start methionine in many bacterial proteins. A recent report revealed the intricate interaction of uL23 with yet another essential player in bacteria, the ATPase SecA, which is best known for its role during post-translational secretion of proteins across the bacterial SecYEG translocon.

Protein targeting and transport processes are generally classified as being either co-translational, i.e. when protein transport is coupled to protein synthesis, or post-translational, i.e. when both processes are uncoupled. Preventing the accumulation of potentially aggregation-prone intermediates is the intrinsic advantage of co-translational transport and therefore bacteria use this pathway primarily for hydrophobic inner membrane proteins. Co-translational protein targeting is initiated by the early recruitment of the signal recognition particle (SRP) to ribosomes synthesizing membrane proteins. But the exact timing of this interaction had been a matter of debate. The initial concept that the N-terminal signal sequence of a membrane protein, also called signal-anchor sequence, had to be fully exposed to the outside of the ribosomal tunnel for SRP to capture it, was questioned by cross-linking and kinetic data primarily provided by the labs of A. Johnson, J. Luirink and W. Wintermeyer. Their data instead suggested that SRP is recruited to ribosomes even before the signal sequence is exposed. Indeed, cryo-EM data from the Ban lab and cross-linking data from our lab, demonstrated that SRP interacts with a β-hairpin-loop of uL23 that is located inside of the ribosomal tunnel, approx. 20 Å away from the tunnel exit. A flexible C-terminal α-helix, which is part of the signal sequence binding domain of SRP, protrudes into the tunnel of non-translating ribosomes, but is sequentially displaced by the growing polypeptide once translation starts. This displacement probably allows SRP to adopt a conformation at the tunnel exit that facilitates signal sequence binding and subsequent targeting of the SRP-ribosome-nascent chain complex to the SRP receptor FtsY, which is bound to the SecYEG translocon. Upon GTP-dependent dissociation of the SRP-FtsY interaction, the ribosome docks onto the SecYEG translocon and successive translation threads the growing membrane protein into the SecYEG channel and subsequently into the lipid phase.

The post-translational mode of protein transport implies that substrates are first completely synthesized and released from the ribosome, before they are captured by the ATPase SecA **(Fig. 1)**. Like FtsY, SecA binds directly to the SecYEG translocon and both FtsY and SecA serve as specific receptor subunits of the SecYEG transport channel. SecYEG-bound SecA will recognize the cleavable signal sequence of secretory proteins, which are destined for the periplasm or the outer bacterial membrane. Despite the presumed post-translational targeting mode, secretory proteins don't stay unprotected during their synthesis, but are in contact with chaperones like Trigger Factor or SecB. Early reports by the Johnson and Müller groups suggesting that SecA can already interact with its substrates while they are synthesized were not met with a lot of enthusiasm. Only after the groups of Huber and Bukau clearly demonstrated that SecA interacts with *E. coli* ribosomes, the possibility of a co-translational substrate recognition by SecA gained momentum. This was the kickoff for our recent analysis revealing almost identical binding modes of SecA and SRP with the *E. coli* ribosome.

**Figure 1:**
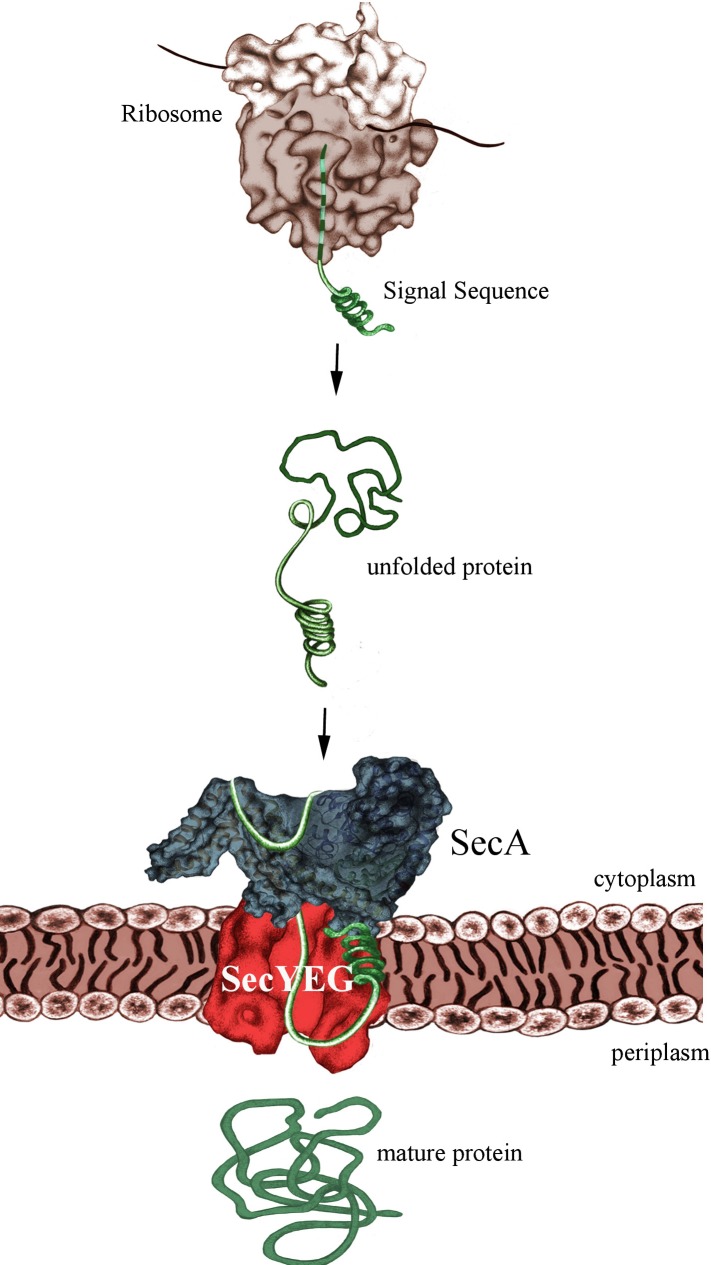
FIGURE 1: SecA-dependent post-translational targeting of secretory proteins to the SecYEG-translocon. For simplicity, chaperone interactions are not shown.

Knüpffer *et al.* employed site-directed *in vitro* cross-linking for determining the SecA-ribosome contact sites. They identified uL23 as primary binding site for SecA on the ribosome and further showed that SecA not only binds to the surface-exposed residues of uL23, but also to the β-hairpin-loop inside of the tunnel **(Fig. 2A)**. This contact involves the N-terminus of SecA and residues of the closely located helical-linker domain. Like SRP, SecA retracts from the tunnel when a nascent protein emerges, but stays in contact with surface-exposed residues of uL23. Thus, SecA obviously scans the ribosomal tunnel for emerging substrates, as previously observed also for SRP. However, neither SRP nor SecA are able to decode the sequence information of the growing polypeptide at this early stage, because retraction of either SecA or SRP occurs independently of the nature of the emerging polypeptide. Still, the early ribosome scanning mode of both SRP or SecA offers several advantages. It gives SRP and SecA a competitive advantage over other uL23 interacting proteins and allows them to shield the emerging signal sequence from the aqueous environment of the tunnel exit and the cytosol. Once sufficient portions of the signal sequences are exposed, SecA and SRP will specifically bind to their client proteins and target them to the membrane for transport.

**Figure 2:**
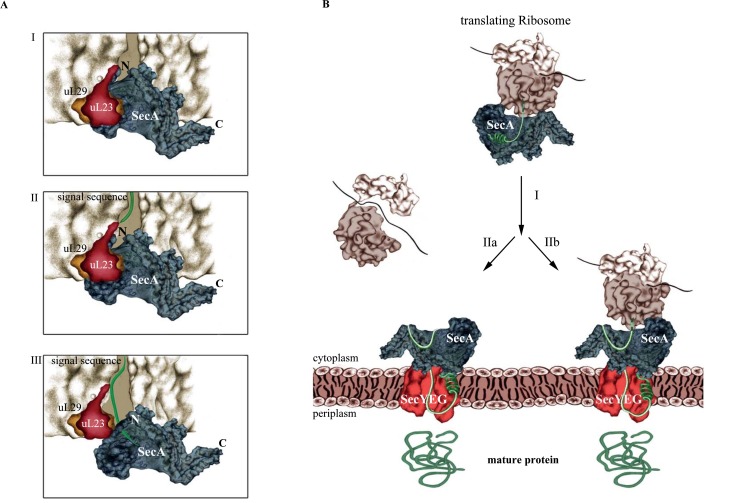
FIGURE 2: SecA-dependent co-translational targeting of secretory proteins to the SecYEG translocon. **(A)** Scanning of the ribosomal tunnel by SecA. I. SecA contacts the intra-tunnel loop of uL23 II. In the presence of an emerging polypeptide, SecA retracts from the tunnel interior. III. SecA traps the signal sequence. N and C correspond to the N-terminus and C-terminus of SecA, respectively. **(B)** Co-translational targeting by SecA. I. SecA targets the nascent secretory protein to the SecYEG channel. At the membrane, SecA is released from the ribosome and binds to the SecYEG translocon. Whether the secretory protein is completely released from the ribosome before translocation starts (IIa) or whether translocation starts already during ongoing translation is unknown (IIb).

The protrusion of SecA's N-terminus into the ribosomal tunnel is intriguing because the N-terminus is also required for the interaction with the SecYEG translocon and with phospholipids. As a consequence, SecA binding to ribosomes or to SecYEG/lipids should be mutually exclusive events. Indeed, Knüpffer *et al.* showed that SecA loses the ability to bind to ribosomes in the presence of *E. coli* membranes. This suggests that the affinity of SecA for the SecYEG translocon is higher than the affinity for ribosomes, which agrees with the observation that *in vivo* the majority of SecA is bound to the membrane and not to ribosomes.

SecA and SRP compete for ribosome binding *in vitro*, but this is likely less relevant *in vivo*, because the concentration of ribosomes is greatly exceeding the concentrations of both SRP and SecA. Furthermore, the high affinity of SecA for SecYEG/lipids further reduces the portion of SecA that is available for ribosome binding. This then raises the question about the physiological relevance of the SecA-ribosome interaction. SecA, FtsY and ribosomes use largely overlapping binding sites on the SecYEG translocon and, as a consequence, are constantly exchanged against each other. SecA that is released from the SecYEG channel can then already scan cytosolic ribosomes and trap – after the signal sequence is exposed - those ribosomes that synthesize secretory proteins. Their targeting to the SecYEG translocon would therefore occur co-translationally **(Fig. 2B)**. Whether the subsequent SecA-dependent translocation across the SecYEG channel is then also a co-translational process or rather occurs post-translationally is currently unknown. Because SecA and ribosomes cannot simultaneously bind to the SecYEG translocon, a co-translational translocation by SecA would still differ from the classical co-translational insertion of membrane proteins, because the ribosome would not be able to bind directly to the SecYEG translocon. However, this does not necessarily mean that translocation can occur only after the substrate is released from the ribosome. It is rather likely that SecA initiates translocation already while the protein is still synthesized. Considering that ATP-dependent translocation is faster than protein synthesis, the translating ribosome would be kept close to the SecYEG-SecA complex. This could even involve simultaneous contacts of SecA to both SecYEG and the translating ribosome (not involving SecA's N-terminus, though).

In a nutshell, the classical concept of Sec-dependent protein transport in bacteria, executed either post-translationally by SecA or co-translationally by SRP, is likely an over-simplification that does not accurately reflect the genuine complexity and flexibility of bacterial protein transport. In addition, these new data further emphasize that the ribosomal tunnel exit serves as a hot-spot for molecular events that determine the fate of newly synthesized proteins.

